# Contextual measurement model and quantum theory

**DOI:** 10.1098/rsos.231953

**Published:** 2024-03-20

**Authors:** Andrei Khrennikov

**Affiliations:** ^1^ International Center for Mathematical Modeling in Physics and Cognitive Sciences, Linnaeus University, 35195 Växjö, Sweden

**Keywords:** contextual measurement model, quantum foundations, contextual probability, generalized probability, quantum instruments, quantum-like modelling

## Abstract

We develop a contextual measurement model (CMM) that is used for the clarification of the quantum foundations. This model matches Bohr’s views on the role of experimental contexts. CMM is based on a contextual probability theory that is connected with generalized probability theory. CMM covers measurements in classical, quantum and semi-classical physics. The CMM formalism is illustrated by a few examples. We consider the CMM framing of classical probability, the von Neumann measurement theory and the quantum instrument theory. CMM can also be applied outside of physics, e.g. in cognition, decision-making and psychology, the so-called quantum-like modelling.

## 1. Introduction

The interrelation of quantum and classical probability theories is a very complex foundational issue involving interpretational, mathematical and philosophical questions. Research in this area is characterized by the diversity of views, opinions and mathematical formalisms (e.g. [1–24]). We remark that, generally, quantum mechanics (QM) is characterized by a diversity of interpretations.

My own understanding is that quantum probability is a machinery for probability update, analogous to classical Bayesian inference [25–34]. In contrast to the latter, quantum probability inference is not based on the Bayes formula for conditional probability. Quantum probability theory is a theory of probability inference with a special class of probability update transformations given by projections or quantum instruments. It is natural to create a general probabilistic framework that covers both the classical and quantum ones. Such generalization can come with a global panorama, as from the top of a mountain, one can enjoy a panorama of the whole city and, through this panorama, connect districts that otherwise look totally separated. In this way, it is easier to find similarities and differences in district plans and the architecture of buildings. As just one of the possible machines for probability update, the quantum probabilistic formalism would lose its mystery.

One such ‘panoramic framework’ is the contextual measurement model (CMM) based on contextual probability space. Its development was initiated in [25], continued in a series of authors’ works (e.g. [26–28]) and summarized in a monograph [29]. In these studies, the main emphasis was on the modification of the formula of total probability (FTP)—its transformation into FTP with an interference term, expressing interference of probabilities, e.g. in the two-slit experiment. In my previous studies, the contextual probability approach was partially shadowed by an appeal to von Mises’ frequency theory of probability [22,30] and the realization of experimental contexts as von Mises collectives.

In this paper, CMM’s development is continued towards the abstract contextual formalization of other basic features of quantum probability, such as order and response replicability effects (RREs) in sequential measurements, entanglement, the violation of the Bell inequalities and establishing coupling with quantum instruments theory as well as with linear space representation (LSR) of generalized probability theory.

CMM is the basis of the *Växjö interpretation* of QM [25,29,31,32]—one of the contextual probabilistic interpretations. Since the probability update is also an information update, the Växjö interpretation is part of the information interpretation of QM. This paper presents CMM consistently in the most general form by highlighting its basic properties, such as interference of probabilities, order effect (OE), entanglement and violation of the Bell inequalities.

The abstract CMM formalism is illustrated by a few examples. We start with the CMM framing of classical probability theory [35,36], which serves as the basis of classical statistical physics and thermodynamics. Then, we consider the von Neumann [1,37] quantum measurement theory with observables given by Hermitian operators and the state update of the projective type and represent it as CMM. The quantum instrument theory is the generalization of the von Neumann theory permitting state updates of the non-projective type, and it can also be represented as CMM. We also show the connection of generalized probability theory with the state space consisting of probability measures with CMM. Finally, LSR for contextual probability space is constructed using the construction dating back to Mackey.

CMM can also be applied outside of physics, in so-called quantum-like modelling (e.g. [38,39]). This is a rapidly developing area of research stimulated by the recent quantum information revolution. In quantum-like modelling, the quantum methodology is applied to cognition, decision-making, psychology, game theory, economics and finance, and AI. Universally, quantum-like models need not be based on the complex Hilbert space formalism. They can employ other contextual probability calculi and CMMs [38].

### 1.1. Contextuality of probability

From the mathematical viewpoint, the essence of the problem lies in generalizations of conditional probability and probability update. On the way to such rethinking of the interrelation between the classical and quantum probability theories, I was strongly influenced by Ballentine who treated all quantum probabilities as conditional probabilities [14,15,18–20]. Later, I learned that this was also Koopman’s viewpoint [4]. It is interesting that Kolmogorov (who in 1933 formalized classical probability in the measure-theoretic framework [35,36]) advertised this conditional viewpoint even for classical probability. This viewpoint was especially clearly described in his early works preceding the monograph [35]. Unfortunately, these works (in Russian and published in proceedings) are practically unknown; see [22] for references and details. I got to know about these ‘pre-axiomatic’ studies of Kolmogorov from Shiryaev and Bulinski, former students of Kolmogorov. But, even monograph [35] ([36]) contains the statement that, in modern words, can be formulated as a statement about the *contextuality of probability*. Kolmogorov’s message is that it is meaningless to speak about probability without determining a complex of experimental conditions and measurement context. This Kolmogorov position matches well with Bohr’s statement on the contextuality of measurement outcomes that is the cornerstone of his complementarity principle [40] (which is better to call the contextuality–complementarity principle [41,42]).

Unfortunately, Kolmogorov’s original message on the contextuality of probability was practically ignored in the further development of classical probability theory. A mathematical work on probability is typically started by fixing one probability measure, without mentioning that it corresponds to some measurement context. The contextuality component of Bohr’s statements on complementarity was not emphasized in quantum foundational research; typically, the Bohr complementarity principle is reduced to the wave–particle duality.

Following Bohr [40], Kolmogorov [35,36], Koopman [4], Accardi [14,15] and Ballentine [17–20], I introduced the contextual probability space and CMM based on it [29]. The main idea behind this approach is to operate solely with contexts and observables and to exclude physical systems from consideration.

A *measurement context* consists of a pre-measurement context C , an observable A and a post-measurement context $C_{A=x}$ corresponding to the outcome A=x , i.e. a triple $\left(C,A,C_{A=x}\right)$ . Transformation $\begin{array}{l} C\to C_{A=x} \end{array}$ can be described as a map $T_{A}(x):C\to C$ , where C is the set of pre-measurement contexts. Alike theory of quantum instruments, we call the pair $I_{A}=(A,T_{A})$ a contextual instrument. The latter is the basic mathematical component of measurement theory. It is meaningless to formulate it solely in terms of observables. The same observable A can be a component of various instruments describing different measurement procedures for A . So, an observable is a theoretical quantity expressing some features of pre-measurement contexts.

In QM, one operates with the notion of ‘state’ not ‘context’. These notions are similar but have some inetrpretational differences (see appendix A for the discussion).

We also mention Feynman’s contextual analysis of the two-slit experiment in [2,3]. He presented the purely probabilistic picture of this fundamental experiment of QM and expressed the interference phenomenon as interference of probabilities. Mathematically, he described this situation as the violation of additivity of probability, the classical formula is disturbed by an additional term, the interference term. In classical probability, the combination of additivity and the Bayes formula for conditional probability leads to FTP, which plays an important role in probability inference. Following this line, Feynman’s conclusion can be rewritten as the violation of classical FTP. The difference between classical and quantum probability models can be moved from the violation of additivity of probability to the violation of the Bayes formula for conditional probability—quantum probability is additive, but conditioning is not Bayesian. The quantum FTP is a perturbation of classical FTP with an additional term—the interference term. The main distinguishing feature of Feynman’s presentation of the two-slit experiment and, generally, quantum interference is its contextual structure. He operates with three contexts $C_{1},C_{2},C_{12}$ : the first slit is open and the second is closed, vice versa, and both slits are open. Quantum probabilistic specialty is expressed not via LSR of states and observables but in a purely contextual probabilistic way.

We make a remark on the notion of contextuality. In the modern quantum information literature, the notion of contextuality is reduced to the contextuality of the joint measurement of a few quantum observables. This sort of contextuality was considered by Bell in [43,44] in his analysis of the violation of the Bell inequalities (although he did not use the term ‘contextuality’; it seems that this term was introduced in the book of Beltrametti and Cassinelli [45]).

Feynman’s contextuality [2,3] is more general and, in fact, coincides with Bohr’s contextuality [40]. In my works including those on the Växjö interpretation, I followed Bohr and Feynman: context as a complex of all physical conditions involved in an experiment.

### 1.2. Linear space versus contextual frameworks for probability

Our foundational pathway is opposite to the pathway leading to the generalized probability theories [5–11,21,45] (see [24] for a review), which is directed towards the creation of general LSR of probability and the measurement process. The LRS also provides a panoramic view that covers both classical and quantum probabilities and observables. This is the linear panorama that illuminates the place of the quantum probability and measurement formalism among other linear models.

We remark that there are several different approaches to generalized probability theory and corresponding measurement theory, but all of them are either equivalent or only slightly different. One of them is the Davies–Lewis [7] operational probability theory grounded on LRS with the base norm spaces, a class of partially ordered linear spaces. The corresponding measurement theory is formulated within instrument theory [7,8,11,12]; in particular, observables are mathematically represented as positive operator-valued measures (POVMs). They are widely used in quantum information theory [46–52] (also see [39,53] for applications to cognition and decision making). Another approach is to start with an abstract definition of state space, a convex subset of a linear space. It goes back to Gudder’s work [9] who constructed the operational representation of quantum states and observables starting with a pre-convex structure. Under natural condition, this approach leads to a convex state space and LRS for the latter. As was shown by Ozawa [11], these two formulations (Davies–Lewis and Gudder) are actually equivalent. In §6, we explore the Davies–Lewis approach for operational LRS of the measurement model with states given by classical probability measures. Then, we express this model in the form of CMM.

The closest to CMM is the model in which one starts with all possible probabilities that can be generated in an experiment, conditioned on preparation and measurement procedures [6]. Then, one proceeds to LSR. This construction can be employed to construct LSR for CMM (§7). However, this is done just to show the connection with previously developed theories.^1^

CMM development is important for quantum foundations. CMM demystifies quantum theory by reducing its probabilistic counterpart to a tool for probability update and inference (cf. with QBism [54–57]); CMM diminishes the role of pure states, in complete agreement with the statistical (ensemble) interpretation of QM; in CMM, quantum interference is just an additive perturbation of classical FTP due to the interplay between a few measurement contexts; the violation of the Bell inequality has the same origin; contextual entanglement is naturally coupled to classical dependence of random variables. The latter demystifies entanglement. This is very important for the resolution of the century-long debate on quantum non-locality; see appendix B for a comparison of CMMs with and without LSRs.

On the other hand, LSR is very convenient from a mathematical viewpoint (simply linear algebra), and operating within the LSR framework is useful in concrete mathematical calculations. However, the calculations should be completed by the critical analysis on the connection of the mathematical LSR constructions with physics. In quantum-like modelling, a similar problem arises—the problem of matching between the output of the Hilbert space formalism and some psychological effects in decision-making [58–62].

Our CMM can be considered the most general probabilistic framework for measurement; in particular, the notion of contextual probability space is based on the first three axioms of Mackey’s theory [6]. Then, Mackey moves towards quantum logic by constraining the model with additional axioms. This path makes theory mathematically elegant but, at the same time, more complex and the basic probabilistic components are blurred by additional mathematical constructions.

## 2. Contextual measurement model

### 2.1. Contextual probability space

**Definition 2.1**. A contextual probability space is a triple *Σ=(C,O,P),* where *C* and *O* are sets of pre-measurement contexts and observables and *P* is the space of the corresponding probability distributions.

In physics, pre-measurement contexts can be associated with preparation procedures.^2^ Each observable A has its range of values $X_{A};$ for simplicity, consider discrete observables, i.e. having finite ranges of values. The following considerations are straightforwardly extended to observables with arbitrary ranges of values. For an observable A and pre-measurement context C, denote the probability of an outcome $x\in X_{A}$ as $P_{C}^{A}(x)\equiv P_{C}(A=x).$ By the definition of a probability distribution,


(2.1) $$P_{C}^{A}(x)\geq 0,\sum_{x\in X_{A}}P_{C}^{A}(x)=1.$$

The range set $X_{A}$ can be endowed with the algebra of all its subsets $F_{A}.$ We set $P_{C}^{A}(G)=\sum_{x\in G}P_{C}^{A}(x),G\in F_{A}.$ This is a probability measure on $F_{A}.$ In the definition of Σ, the symbol P denotes the collection of such probability measures,


$$𝒫=\left\{P_{C}^{A}:C\in 𝒞,A\in 𝒪\right\}$$

(see Axiom 1 in [6]). Elements of P are called contextual probabilities. These are the analogues of the conditional probabilities in the classical (Kolmogorov [36]) probability model. But, we reserve the term ‘conditional probability’ for a special class of contextual probabilities generated by context updates.

It is natural to assume (see Axiom 2 in [6]) that two observables having the same probability distribution for all contexts should coincide, i.e.


(2.2) $$P_{C}^{A_{1}}=P_{C}^{A_{2}}\;\text{ for any }C\in C\Rightarrow A_{1}=A_{2}.$$

We also assume (see Axiom 2 in [6]) that two contexts having the same probability distribution for all observables should coincide, i.e.


(2.3) $$P_{C_{1}}^{A}=P_{C_{2}}^{A}\;\text{ for any }A\in O\Rightarrow C_{1}=C_{2}.$$

The average of an observable A∈O (with $X_{A}\subset R)$ with respect to a pre-measurement context C∈C is defined as


(2.4) $$\langle A\rangle_{C}\equiv E[A|C]=\sum_{x\in X_{A}}xP_{C}(A=x).$$

### 2.2. Context update and conditional probability

Measurement of an observable A with the concrete outcome x in a pre-measurement context C updates this pre-measurement context:


(2.5) $$C\to C_{A=x}.$$

In terms of preparation procedures, we can consider a measurement as a subsequent preparation procedure; context $C_{A=x}$ is the measurement of A and filtering with respect to the fixed outcome x. ^3^ It is natural to consider this map only for contexts belonging to the set


(2.6) $$C_{A}(x)=\{C:P_{C}(A=x)>0\}.$$

If $P_{C}(A=x)=0,$ then the post-measurement context is not well defined. Thus, each observable A and its outcome x determine a map


(2.7) $$T_{A}(x):C\to C,\;C\to C_{A=x}=T_{A}(x)C,$$

with the domain of definition $C_{A}(x).$

The delicate point of measurement theory is that generally an observable does not determine the context update map unequally. An observable A can be measured via different measurement procedures, and each procedure generates its own context update map. A pair $I_{A}$ = (observable, context update map) = ( $A,T_{A})$ is called a *contextual instrument* (cf. §5); a pair $\left(C,I_{A}\right)$ or a triple $\left(C,A,T_{A}\right)$ is called a *measurement context*. We stress once again that a variety of instruments can be associated with the same observable $A:\;I_{A}=(A,T_{A}),I_{A}^{\prime }=(A,T_{A}^{\prime }),I_{A}^{\prime \prime }=(A,T_{A}^{\prime \prime }),....\;.$ We stress that all these update maps have the same domain of definition determined by the observer A ; see equation (2.6).

Typically, one fixes some class of context update maps. In the von Neumann [1,37] measurement theory (§4), this is the class of normalized projections. In quantum instrument theory (§5), these are quantum channels, or more generally, in the theory of Davis–Levis instruments, these are positive trace-preserving maps.

We emphasize that von Neumann’s measurement theory is very special: here, by fixing an observable and a Hermitian operator $\hat{A},$ we automatically fix the update map—via operator’s spectral family. This special situation leads to the illusion that an observable determines the update map. We repeat that generally, this is not the case.

**Definition 2.2**. Let *Σ=(C,O,P)* be a contextual probability space. A CMM is a pair *M=(Σ,I),* where *Σ* is a contextual probability space and *I* is a collection of contextual instruments.

CMM is a set of measurement contexts, i.e. triples $(C,A,T_{A}).$ CMM generates the notion of the conditional probability:

**Definition 2.3**. Consider a measurement context $(C,A,T_{A}).$ Let it generate the output A=x and the corresponding context update, $C\to C_{A=x}=T_{A}(x)C.$ Consider the measurement of another observable B under the condition A=x, i.e. with respect to the context $C_{A=x}.$ The conditional probability is given by the formula


(2.8) $$P_{C,I_{A}}(B=y|A=x)\equiv P_{C_{A=x}}(B=y)=P_{T_{A}(x)C}(B=y),\;C\in C_{A}(x).$$

We note that this definition involves context update only for the A observable; different contextual instruments $I_{A},I_{A}^{\prime },...,$ induce their own probabilistic conditioning, $P_{C,I_{A}}(B=y|A=x),P_{C,I_{A}^{\prime }}(B=y|A=x),...\;.$ For simplicity, we shall typically omit the index $I_{A}$ of dependence on the concrete instrument.

### 2.3. Contextual formula of total probability

Now, we point out that generally, contextual probability differs from the classical Kolmogorov probability [36]. One of the basic classical laws of probability is the law of total probability formulated in the form of FTP:


(2.9) $$P(B=y)=\sum_{x\in X_{A}}P(B=y|A=x)P(A=x).$$

In classical probability theory, contextual probability is identified with the conditional one (§3), and the contextual-conditional analogue of FTP has the form


(2.10) $$P_{C}(B=y)=\sum_{x\in X_{A}}P_{C}(B=y|A=x)P_{C}(A=x);$$

see equation (3.4). However, in a general contextual probability space, this formula can be violated:


(2.11) $$P_{C}(B=y)\neq \sum_{x\in X_{A}}P_{C}(B=y|A=x)P_{C}(A=x)=$$

$$\sum_{x\in X_{A}}P_{C_{A=x}}\,\left(B=y\right)\,P_{C}\,\left(A=x\right).$$

The difference between the left-hand side (l.h.s.) and right-hand side (r.h.s.) determines the degree of context disturbance due to its update; it can serve as a measure of nonclassicality of a contextual model:


(2.12) $$\delta_{C}(B=y|A)=P_{C}(B=y)-\sum_{x\in X_{A}}P_{C}(B=y|A=x)P_{C}(A=x).$$

We call this quantity *the interference term* [25,29]. This consideration can be formalized in the contextual FTP (with an interference term):


(2.13) $$P_{C}(B=y)=\sum_{x\in X_{A}}P_{C}(B=y|A=x)P_{C}(A=x)+\delta_{C}(B=y|A).$$

The equality of the interference term to zero is a necessary condition of the classical probabilistic representation of a contextual probability model, but it is not a sufficient condition [29].

Let us, for the moment, jump to §4, where the von Neumann quantum measurement model is treated as CMM. Consider dichotomous observables $A=x_{1},x_{2}$ and $B=y_{1},y_{2}.$ In this case, the interference term has the form


$$\delta_{C}\,\left(B=y|A\right)=$$

(2.14) $$2\cos\theta \sqrt{P_{C}(B=y|A=x_{1})P_{C}(A=x_{1})P_{C}(B=y|A=x_{2})P_{C}(A=x_{2})},$$

where context C is identified with the quantum state ψ (for simplicity, consider contexts corresponding to pure states) and the angle θ=θ(B=y|A;ψ). For dichotomous observables, even in general CMM, it is useful to write the interference term as


$$\delta_{C}\,\left(B=y|A\right)=$$

(2.15) $$2\lambda \sqrt{P_{C}(B=y|A=x_{1})P_{C}(A=x_{1})P_{C}(B=y|A=x_{2})P_{C}(A=x_{2})},$$

where λ=λ(B=y|A;C). If |λ|≤1, then this is the trigonometric interference, and the interference term has the form of equation (2.14). If |λ|≥1, then this is the hyperbolic interference, and the interference term can be represented as


$$\delta_{C}\,\left(B=y|A\right)=$$

(2.16) $$\pm 2\cosh\sqrt{P_{C}(B=y|A=x_{1})P_{C}(A=x_{1})P_{C}(B=y|A=x_{2})P_{C}(A=x_{2})}.$$

In the quantum framework, such interference can be generated by quantum instruments [63]. In general CMM, we can employ the hyperbolic version of QM [29].

### 2.4. Conditional joint probability distribution and order effect

For observables $A_{1},A_{2}\in O,$ the conditional joint probability distribution (JPD) is defined by


(2.17) $$P_{C}(A_{1}=x_{1},A_{2}=x_{2})=P_{C}(A_{1}=x_{1})P_{C}(A_{2}=x_{2}|A_{1}=x_{1}).$$

We remark that this is really a probability distribution, i.e. $\sum_{x_{1},x_{2}}P_{C}(A_{1}=x_{1},A_{2}=x_{2})=1.$ We can also define JPD for inverse order of measurements, $P_{C}(A_{2}=x_{2},A_{1}=x_{1})=P_{C}(A_{2}=x_{2})P_{C}(A_{1}=x_{1}|A_{2}=x_{2}).$ Observables $A_{1},A_{2}$ show OE with respect to context C, if


(2.18) $$P_{C}(A_{1}=x_{1},A_{2}=x_{2})\neq P_{C}(A_{2}=x_{2},A_{1}=x_{1}),$$

for at least one pair of outcomes $(x_{1},x_{2});$ otherwise, there is no OE in context C.

We remark that OE was actively investigated in decision-making and psychology, both theoretically and experimentally; in particular, within quantum-like modelling—the applications of quantum methodology and formalism to decision-making and psychology [58–62].

### 2.5. Conditional compatibility

In the absence of OE, we have


(2.19) $$P_{C}(A_{1}=x_{1},A_{2}=x_{2})=P_{C}(A_{2}=x_{2},A_{1}=x_{1}),\;x_{j}\in X_{A_{j}},$$

i.e.


(2.20) $$P_{C}(A_{1}=x_{1})P_{C}(A_{2}=x_{2}|A_{1}=x_{1})=P_{C}(A_{2}=x_{2})P_{C}(A_{1}=x_{1}|A_{2}=x_{2}).$$

In this case, we call the observables *conditionally compatible* for context C∈C , and their JPD is defined by equation (2.19). We remark that the marginals of JPD coincide with the probability distributions $P_{C}^{A_{i}}.$

In the von Neumann CMM $M_{QVN}$ (§4) with observables and state updates of the projection type, conditional compatibility for all possible pre-measurement contexts (given by density operators) is equivalent to the commonly considered compatibility of observables and their representation by commutative Hermitian operators.

By considering conditional JPD, we do not assume that the observables $A_{1}$ and $A_{2}$ are jointly measurable. We consider sequential measurements, say first $A_{1}$ then $A_{2}$ or vice versa. We remark that, in fact, precisely, this experimental setup is realized in the Bell experiments. Here, the instances of time for the measurement’s outputs on subsystems coincide with zero probability; always the click of the photo-detector for the subsytem $S_{1}$ is before the click of the photo-detector for the subsystem $S_{2}$ or vice versa, and the time window serves for click pairing.

Conditional compatibility implies the Bayes formula for the conditional probability:


(2.21) $$P_{C}(A_{2}=x_{2}|A_{1}=x_{1})=\frac{P_{C}(A_{1}=x_{1},A_{2}=x_{2})}{P_{C}(A_{1}=x_{1})},$$

(2.22) $$P_{C}(A_{1}=x_{1}|A_{2}=x_{2})=\frac{P_{C}(A_{1}=x_{1},A_{2}=x_{2})}{P_{C}(A_{2}=x_{2})}.$$

The equality given in equation (2.20) implies the Bayes theorem for probability inference. Let the outcomes of the observable $A_{2}$ label some hypotheses, $H_{1},...,H_{m}.$ Then, equation (2.20) is written as


(2.23) $$P_{C}(A_{2}=H_{j}|A_{1}=x_{1})=\frac{P_{C}(A_{2}=H_{j})P_{C}(A_{1}=x_{1}|A_{2}=H_{j})}{P_{C}(A_{1}=x_{1})}.$$

The Bayes formula for conditional probability equations (2.21) and (2.22) implies the validity of the classical FTP, i.e. the Bayes theorem can be written in the standard form:


(2.24) $$P_{C}(A_{2}=H_{j}|A_{1}=x_{1})=\frac{P_{C}(A_{2}=H_{j})P_{C}(A_{1}=x_{1}|A_{2}=H_{j})}{\sum_{H_{i}}P_{C}(A_{1}=x_{1}|H_{i})P_{C}(A_{2}=H_{i})}.$$

### 2.6. Replicability and response replicability

Observable A shows *replicability* for the context C, if


(2.25) $$P_{C}(A=x,A=x)=P_{C}(A=x),$$

or


(2.26) $$P_{C_{A=x}}(A=x)=1.$$

Observable A shows replicability if equation (2.25) holds for any $C\in C_{A}(x),x\in X_{A}.$

In quantum-like modelling, the following effect plays an important role. Observables $A_{1}$ and $A_{2}$ show RRE with respect to context C, if


(2.27) $$P_{C}(A_{1}=x_{1},A_{2}=x_{2},A_{1}=x_{1})=P_{C}(A_{1}=x_{1},A_{2}=x_{2}),$$

(2.28) $$P_{C}(A_{2}=x_{2},A_{1}=x_{1},A_{2}=x_{2})=P_{C}(A_{2}=x_{2},A_{1}=x_{1})$$

for all pairs of outcomes $(x_{1},x_{2}).$ This is a kind of memory effect. The challenging problem for quantum-like modelling was the combination of OE and RRE [60]. It was solved in articles [61,62] within quantum instrument theory.

### 2.7. Correlations and Bell-type inequalities

Consider a pair of conditionally compatible observables A,B∈O (with $X_{A},X_{B}\subset R).$ Their correlation with respect to a context C∈C is defined as


(2.29) $$\langle AB\rangle_{C}\equiv E[AB|C]=\sum_{x,y}xyP_{C}(A=x,B=y).$$

The most popular Bell-type inequality is the CHSH inequality. We consider this inequality within CMM. There are given four observables $A_{i},B_{j},i,j=1,2,$ valued in [−1,1]; observables in the pairs $\left(A_{i},B_{j}\right)$ are conditionally compatible for some context C with JPDs $P_{C}^{A_{i},B_{j}}.$ The CHSH inequality has the form


(2.30) $$|\langle A_{1}B_{1}\rangle_{C}+\langle A_{2}B_{1}\rangle_{C}+\langle A_{1}B_{2}\rangle_{C}-\langle A_{2}B_{2}\rangle_{C}|\leq 2,$$

i.e.


(2.31) $$|\sum_{x,y}xy(P_{C}^{A_{1},B_{1}}(x,y)+P_{C}^{A_{2},B_{1}}(x,y)+P_{C}^{A_{1},B_{2}}(x,y)-P_{C}^{A_{2},B_{2}}(x,y))|\leq 2.$$

If there exists a probability measure $P_{C}$ such that JPDs $P_{C}^{A_{i},B_{j}}$ can be obtained as its marginals, e.g.


$$P_{C}^{A_{1},B_{1}}\,\left(x,y\right)=\sum_{x_{2},y_{2}}P_{C}\,\left(x,x_{2},y,y_{2}\right),$$

then the CHSH inequality holds true. However, if such $P_{C}$ does not exist, then this inequality can be violated, and the maximum of its l.h.s. with respect to contexts C∈C and observables $A_{1},A_{2},B_{1},B_{2}\in O$ valued in [−1,1] can approach the value of 4, maxCHSH=4. This maximum depends on CMM. For von Neumann CMM $M_{QVN},$ this is $\max CHSH=2\sqrt{2}.$

### 2.8. Functions of observables

Suppose that all observables are valued in multidimensional real space, and we remove (for the moment) the restriction that observables’ ranges of values are finite. We consider probability measures on the σ -algebra B of the Borel sets, which is generated by all semi-open intervals, $(\alpha_{1},\beta_{1}]\times \cdots \times (\alpha_{n},\beta_{n}].$ So, $P_{C}^{A}$ is a probability measure on B. A function $f:R^{n}\to R^{m}$ is called B -measurable if for any Borel subset G of $R^{m}$ its preimage $f^{-1}(G)$ is a Borel subset of $R^{n}.$ Only such functions are considered.

Following Mackey [6] (Axiom 3), we assume that for each A∈O with the range of values $R^{n}$ and a Borel function $f:R^{n}\to R^{m}$ , there exists $B=B_{f}\in O$ such that, for any C∈C and Borel set $G\subset R^{m},$


(2.32) $$P_{C}^{B}(G)=P_{C}^{A}(f^{-1}(G)).$$

Such observable is uniquely defined, due to condition (2) (Mackey’s Axiom 2), and it can be denoted as B=f(A).

Two observables $A_{1}$ and $A_{2}$ are called functionally compatible (jointly measurable) if there exists an observable A and functions $f_{i}$ such that $A_{i}=f_{i}(A).$ For the von Neumann CMM $M_{QVN}$ (§4), functional compatibility is equivalent to compatibility and, hence, conditional compatibility. Generally, in CMM, the interrelation between these two notions is complex, and we shall not proceed to a detailed comparison.

### 2.9. Entanglement of contextual instruments

Entanglement is typically considered as one of the distinguishing features of LSR; from my viewpoint, the association of entanglement with LSR and the tensor product structures shadow its physical nature; its mathematical description is identified with physics. As was shown in the articles [64,65], the notion of entanglement can be formalized in the purely probabilistic framework and dissociated from the tensor product and generally from LSR.

By starting with such a probabilistic approach to the notion of entanglement, the authors of [64,65] proceed towards its complex Hilbert space realization. Now, we present the purely probabilistic picture of entanglement. The main value of the contextual probabilistic realization of entanglement is in the clarification of its foundational meaning. At the same time, the use of LSR can essentially simplify concrete calculations. However, one should be careful with the connection of the mathematical structures of LSR with physics (or in quantum-like modelling such as psychology and decision-making).

Consider CMM M=(Σ,I), where Σ=(C,O,P) is a contextual probability space and I is a collection of contextual instruments of this model, i.e. pairs (observable, state update map). Consider two contextual instruments $I_{A}=(A,T_{A})$ and $I_{B}=(B,T_{B}).$

**Definition 2.4**. In pre-measurement context *C∈C,* the outcome *B=β* depends on the outcomes of *A* if for at least two values of *$A,\alpha =\alpha_{i},\alpha_{j},$* the corresponding conditional probabilities do not coincide:


(2.33) $$P_{C}\left(B=\beta |A=\alpha_{i}\right)\neq P_{C}\left(B=\beta |A=\alpha_{j}\right)$$

Thus, the probability to get the outcome B=β if the preceding A -measurement had the outcome $A=\alpha_{i}$ differs from the probability to get the same outcome B=β if the preceding A -measurement had the outcome $A=\alpha_{j}.$ We remark that the update map $T_{B}$ is not involved in this definition, i.e. one can consider just an observable B without referring to the corresponding instrument $I_{B}.$ For symmetry reason, we consider two instruments.

We note that the outcome B=β does not depend on the outcomes of the observable A if


(2.34) $$P_{C}(B=\beta |A=\alpha_{i})=P_{C}(B=\beta |A=\alpha_{j}),\;\text{for all pairs}\;\alpha_{i},\alpha_{j},$$

i.e. the conditional probability for this outcome is constant with respect to the outcomes of A. Denote it $P_{C}(B=\beta |A).$ The following natural question arises. Does the probability $P_{C}(B=\beta |A)$ coincide with the unconditional probability $P_{C}(B=\beta )?$

**Definition 2.5**. Two instruments *$I_{A}$* and *$I_{B}$* are called *AB *-entangled in *C∈C* or *C* is *AB *-entangled, if all outcomes of the *B *-observable depend on outcomes of the *A *-observable, i.e. for all *β* condition (2.33) holds for some *$\alpha_{i},\alpha_{j}.$*

Concerning the notation ‘ AB -entangled’, it would be better to write ‘ $I_{A}I_{B}$ -entangled’ to emphasize that this is the entanglement of instruments and not simply the observables but to make the notation compact, we proceed with ‘ AB -entangled’. The order of observables is important. Generally, AB -entanglement does not imply BA -entanglement. This is a purely probabilistic definition that does not involve LSR and can be applied to any statistical physical theory. This definition formalizes the dependence of observables. We introduce the following quantitative measure of entanglement.

**Definition 2.6**. For contextual instruments *$I_{A}$* and *$I_{B}$* and pre-measurement context *C, AB* -concurrence of conditional probabilities is defined as


(2.35) $$\lambda_{AB}(\psi )=\sum_{\beta }\sum_{\alpha \neq \alpha^{\prime }}|P_{C}(B=\beta |A=\alpha )-P_{C}(B=\beta |A=\alpha^{\prime })|.$$

The crucial issue is that AB -concurrence depends on a pair of instruments.

**Proposition 2.7**. *For dichotomous observables A,B=±, dependencies of the values B=− and B=+ on the outcomes of A are equivalent. Thus, each dependence is equivalent to the AB -entanglement*.

**Proof**. In the state context C, the value B=− depends on the outcomes of A if


(2.36) $$P_{C}(B=-|A=+)\neq P_{C}(B=-|A=-).$$

This automatically implies that even the value B=+ depends on the outcomes of A,


$$P_{C}\left(B=+|A=+\right)=1-P_{C}\left(B=-|A=+\right)\neq$$

$$1-P_{C}\left(B=-|A=-\right)=P_{C}\left(B=+|A=-\right),$$

i.e. instruments $I_{A}$ and $I_{B}$ are entangled in the context C.

As was already pointed out, in articles [64,65], contextual probabilistic entanglement can be realized in the complex Hilbert space and, in this way, connected with the ordinary notion of entanglement. In the LSR representation, the main distinguishing feature of AB -entanglement (definition 2.5) is that it is associated with a pair of instruments, $I_{A},I_{B}.$ The standard definition of entanglement is coupled with the tensor product structure and not with two concrete instruments (observables).

For simplicity, let us consider CMM $M_{QVN}$ (§4) with von Neuman observables [37]; in this CMM, an observable $\hat{A},$ a Hermitian operator, automatically determines the state update map through its spectral family. So, there is no need to operate with instruments; one can solely operate with observables. In this CMM (which is typically used in entanglement studies), contextual probabilistic entanglement is associated with the pairs of observables, i.e. two observables are entangled or disentangled in some state (context) $C=\hat{\rho },$ where $\hat{\rho }$ denotes a density operator. The main mathematical features of AB -entanglement and ordinary tensor product-based entanglement are similar, but some essential differences can be found [64,65].

The probabilistic viewpoint on the ‘EPR-paradox’ [66] is presented in Schrödinger’s papers [67,68], which initiated the modern theory of entanglement. However, this theory ignores the important message of Schrödinger: entanglement characterizes the probability update for the outcomes of observable B conditioned on the outcomes of observable A. In the framework of [67,68], it is meaningless to speak about the entanglement without specifying the observables. The state update—the Hilbert space representation of the probability update—encodes the procedure of conditional prediction. For Schrödinger, quantum formalism is a mathematical machinery for probability prediction (as in the Växjö interpretation or QBism), and a quantum state is a part of such machinery. We can say that Schrödinger interpreted quantum probabilities as conditional (contextual) probabilities and entanglement as contextual probabilistic entanglement. However, this is my private interpretation of Schrödinger’s views, and many experts in quantum foundations may disagree with me.

By following Schrödinger [67,68], in article [64], we considered a special sort of contextual probabilistic entanglement that matches perfectly with the Schrödinger analysis of the EPR argument.

**Definition 2.8**. For *C∈C,* instruments *$I_{A}$* and *$I_{B}$* are perfectly conditionally correlated for values *(A=α,B=β)* if the conditional probability to get the outcome *B=β* and if the preceding *A *-measurement had the outcome *A=α* equal to 1:


(2.37) $$P_{C}(B=\beta |A=\alpha )=1.$$

More generally, consider observables with values $\left(\alpha_{i}\right)$ and $\left(\beta_{i}\right)$ and some set Γ of pairs $(\alpha_{i},\beta_{j}).$

**Definition 2.8a** (EPR entanglement). Let *C∈C.* If instruments *$I_{A}$* and *$I_{B}$* are perfectly conditionally correlated for all pairs belonging to *Γ,* then they are called EPR-entangled with respect to set *G* in the context *C.*

We are interested in sets Γ such that each of α and β values appears in the pairs once and only once. We call such EPR entanglement complete.

For example, for two dichotomous observables with α,β=±, we consider, e.g. the set of the pairs (A=+,B=−),(A=−,B=+), in short, A=−B EPR entanglement or the pairs (A=+,B=+),(A=−,B=−),A=B EPR entanglement. We analyse such EPR entanglements.

Let us start with A=−B EPR entanglement, i.e. $P_{C}(B=-|A=+)=1$ and $P_{C}(B=+|A=-)=1.$ Thus, $P_{C}(B=+|A=+)=0$ and $P_{C}(B=-|A=-)=0,$ and $P_{C}(B=-|A=+)=1\neq P_{C}(B=-|A=-)=0$ and $P_{C}(B=+|A=-)=1\neq P_{C}(B=+|A=+)=0.$ In this case, EPR-entangled instruments are automatically entangled in the sense of definition 2.5.

Now turn to A=B EPR entanglement, i.e. $P_{C}(B=+|A=+)=1$ and $P_{C}(B=-|A=-)=1.$ Thus, $P_{C}(B=-|A=+)=0$ and $P_{C}(B=+|A=-)=0,$ and $P_{C}(B=+|A=+)=1\neq P_{C}(B=+|A=-)=0$ and $P_{C}(B=-|A=-)=1\neq P_{C}(B=-|A=+)=0.$ And again, EPR-entangled instruments are automatically entangled in the sense of definition 2.5.

Thus, EPR entanglement is just a very special case of AB -entanglement.

For dichotomous observables, the AB -concurrence of conditional probabilities, equation (2.35), has the form $C≀\setminus_{AB}(\psi )=|P(B=+|A=-)-P(B=+|A=+)|+|P(B=-|A=-)-P(B=-|A=+)|,$ and hence it can be written as


(2.38) $$\lambda_{AB}(\psi )=2|P(B=+|A=-)-P(B=+|A=+)|.$$

From this formula, we immediately obtain the following characterization of maximally AB -entangled states:

**Proposition 2.9**. AB *-concurrence of conditional probabilities approaches its maximal value, $\lambda_{AB}(\psi )=2,$ if and only if the instruments are EPR-entangled in the pre-measurement context C.*

### 2.10. Distinguishing features of contextual measurement models

We list the probabilistic constraints that can be used to distinguish different CMMs (theoretically and experimentally):

– violation of FTP
– OE
– RRE
– OE+RRE
– violation of Bell inequalities

### 2.11. Interpretations of contextual probability

Probability is characterized by the diversity of interpretations [22]. We now discuss the interpretations of contextual probability. We start with the remark that mathematically, a contextual probability space Σ=(C,O,P) cannot be described as single Kolmogorov probability space: Σ is a bunch of such spaces. However, fixed C∈C and A∈O can be realized within some probability space K=(Ω,F,P) with the realization of observable A by a random variable $a:\Omega \to X_{A};$ its probability distribution coincides with $P_{C}^{A},$ i.e.


$$P_{C}^{A}\left(\alpha \right)=P\left(\omega \in \Omega :a\left(\omega \right)=\alpha \right),\alpha \in X_{A}.$$

We note that a contextual probability space Σ can be represented as


(2.39) $$\Sigma =\cup_{C,A}K_{C,A},$$

where $K_{C,A}$ is a Kolmogorov probability space for describing the measurement of the observable A in the pre-measurement context C.

Therefore, one can assign any interpretation used for probability defined in the measure-theoretic framework to the contextual probability. The main interpretation employed in classical and quantum physics is the frequency interpretation. In the Kolmogorov theory [35,36], this interpretation is mathematically rooted to the strong law of large numbers. Another basic interpretation of probability in physics is the statistical (ensemble) interpretation. By this interpretation, $\Omega =\Omega_{C}$ represents an ensemble of systems prepared for measurement, and the probability measure $P=P_{A}$ depends on the observable A. Finally, we mention the subjective interpretation. It is widely employed in decision-making and psychology but was not used in physics until QBism was invented.

In the contextual probability, we need not represent a pre-measurement context C by an ensemble of systems. Instead, we can consider a sequence of measurements of an observable A in the same pre-measurement context *C*,


(2.40) $$x\equiv x_{C,A}=(x_{1},...,x_{N},...,...),$$

where $x_{j}(\in X_{A}=\{\alpha_{1},...,\alpha_{m}\})$ are the measurement’s outcomes. Such sequence determines the frequencies of realizations of the concrete values,


(2.41) $$\nu_{N}(\alpha_{j})=n_{N}(\alpha_{j})/N,$$

where $\nu_{N}(\alpha_{j})$ is the number of the measurement’s outcomes with the fixed value $\alpha_{j}.$ The probability to obtain the value $\alpha_{j}$ in a sequence of measurements x is defined as the limit


(2.42) $$P_{C}^{A}(\alpha_{j})\equiv \lim_{N\to \infty }\nu_{N}(\alpha_{j}).$$

This is the straightforward frequency introduction of probability. The deep mathematical theory of frequency probability was developed by von Mises [30] (see also [22] for an introduction). A sequence x generated by observations is called a *collective*. Von Mises’ theory is a theory of collectives. Instead of operations on sets, as done in the Kolmogorov measure-theoretic theory, von Mises constructed a probability theory based on operations with collectives. We remark that $P_{C}(A=\alpha_{j})\equiv P_{C}^{A}(\alpha_{j})$ can be considered as the probability generated by the collective $x\equiv x_{C,A},$ i.e. $P_{C}^{A}(\alpha_{j})=P_{x_{C,A}}(\alpha_{j}).$ It is important to note that not all collectives are compatible or combinable in von Mises’ terminology. Two collectives x and y are combinable if their combination


(2.43) $$z=(z_{1},...,z_{N},...),\;z_{j}=(x_{j},y_{j}),$$

is also a collective, and the probability distributions $P_{x}$ and $P_{y}$ are marginal for the probability distribution $P_{z},$ i.e.


(2.44) $$P_{x}(\alpha_{j})=\sum_{\beta_{i}}P_{z}(\alpha_{j},\beta_{i}),\;P_{z}(\beta_{i})=\sum_{\alpha_{j}}P_{z}(\alpha_{j},\beta_{i}).$$

The frequency probability theory contains the notion of conditional probability that is similar to CMM’s conditioning, and the post-measurement context $C_{A=a}$ corresponds to the post-measurement collective $x_{C,A=a}$ (see [22,30] for details).

In contrast to the Kolmogorov measure-theoretic probability theory, in the von Mises frequency probability theory, classical FTP is violated [22,29], and the probabilities can interfere and generate the additive perturbation of FTP in the form of the interference term. The presence of incombinable collectives leads to the violation of the Bell-type inequalities [22,29].

The notion of a collective was the seed for the future growing theory of random sequences. Besides the existence of the limits for frequencies, equation (2.42), a collective is characterized by the stability of these limits with respect to place selections within a sequence x , i.e. the limit probability is the same for all subsequences of x for a special class of place selections. However, von Mises’ definition of place selection was criticized for non-rigorousness. Its critical analysis was very fruitful and led to the modern theory of randomness (e.g. [69]). In particular, the monograph presents a ‘light version’ of the von Mises theory. In physics (at least in quantum physics), one does not analyse in the random structure of the sequences of the measurement’s outcomes. In principle, in QM, one can proceed with ‘light-collectives’ determined solely by the existence of limits (2.42). The calculus of such ‘light-collectives’ can be explored for the description of the probabilistic structure of QM [69]. The first version of the contextual probability was presented in such a ‘light-frequency’ framework.

Finally, we note that von Neumann [1,37] pointed to the von Mises [30] frequency probability as the probabilistic foundation for QM. This is a complex foundational issue.

## 3. Contextual measurement model for Kolmogorov theory

Let K=(Ω,F,P) be a Kolmogorov probability space [36]. Here, Ω is a set of any origin, F is a collection of its subsets forming σ -algebra, i.e. F is closed with respect to countable unions and intersections and the operation of complement. (If Ω is finite, then F is a collection of all its subsets and P is a probability measure on F. )

Set C={C∈F:P(C)≠0}. This is the set of contexts. For each context C, the Bayes formula defines the conditional probability measure


$$P_{C}\,\left(G\right)=P\,\left(G\cap C\right)/P\,\left(C\right),G\in ℱ.$$

We highlight that the statistical mixtures of contexts are not determined, i.e. for subsets $C_{1},C_{2}$ of Ω and weights $p_{1},p_{2}\geq 0,p_{1}+p_{2}=1,$ there is no subset C of Ω that can be identified with the weighted sum $p_{1}C_{1}+p_{2}C_{2}.$

As an illustrative example, consider some agricultural region Ω , and as contexts, consider its sub-fields (some areas). Generally, there is no field of the form $p_{1}C_{1}+p_{2}C_{2}.$ In applications to decision-making and cognition, one can meet the situations such that $p_{1}C_{1}+p_{2}C_{2}$ is determined only for a few pairs of weights $p_{1},p_{2}.$ This situation is related to the poorness of the set of possible experimental contexts.

The set of observables O is the set of (discrete) random variables, $a:\Omega \to X_{a},$ where $X_{a}$ is a finite set (discrete random variables are considered for simplicity). Denote this set by the symbol $R_{d}.$ For $x\in X_{a},$ we set $\Omega_{a=x}=\{\omega \in \Omega :a(\omega )=x\}.$ The contextual probability coincides with the conditional probability given by the Bayes formula:


(3.1) $$P_{C}^{a}(x)\equiv P_{C}(a=x)=P(\Omega_{a=x}\cap C)/P(C).$$

Thus, the set of probability distributions $P=\{P_{C}^{a}:C\in C,a\in O\}.$

For any set D∈F and random variable $a\in R_{d},x\in X_{a},$ we define that set


$$D_{a=x}=\{\omega \in D:a(\omega )=x\}=D\cap \Omega_{a=x}$$

and the map


(3.2) $$T_{a}(x):F\to F,\;D\to D_{a=x}=T_{a}(x)D.$$

For any context C∈C and random variable $a\in R_{d},x\in X_{a},$ we define the family of contexts


$$C_{a}(x)=\{C\in C:P_{C}(a=x)>0\}=\{C\in C:P(C_{a=x})>0\}.$$

Each random variable a and its outcome x determine a map


(3.3) $$T_{a}(x):C\to C,\;C\to C_{a=x}=T_{a}(x)C,$$

with the domain of definition $C_{a}(x).$

Thus, classical CMM $M_{cl}$ consists of measurement contexts composed of pre-measurement contexts—elements of F with non-zero probabilities, observables—(discrete) random variables and context update maps, $T=\{T_{a}(x)\}.$ Here, each observable a, random variable, determines uniquely the context update maps $T_{a}(x),$ and, hence, the contextual instrument.

The conditional probability is given by the Bayes formula:


$$P_{C}\,\left(b=y|a=x\right)=P\,\left(\Omega_{b=y}\cap \Omega_{a=x}\cap C\right)/P\,\left(\Omega_{a=x}\cap C\right)$$

$$=P\,\left(\Omega_{b=y}\cap C_{a=x}\right)/P\,\left(C_{a=x}\right)=P_{C_{a=x}}\,\left(b=y\right).$$

Since, for each $C\in C,P_{C}$ is a probability measure, for any pair of random variables a,b , we have the following version of FTP (§2.3):


(3.4) $$P_{C}(b=y)=\sum_{x\in X_{a}}P_{C}(b=y|a=x)P_{C}(a=x)=$$

$$\sum_{x\in X_{a}}P_{C_{a=x}}\,\left(b=y\right)\,P_{C}\,\left(a=x\right).$$

In this measurement model, all observables are compatible, conditional JPD coincides with JPD (again by Bayes formula); no pair of observables show OE, since


$$P_{C}\,\left(a_{1}=x_{1},a_{2}=x_{2}\right)=P_{C}\,\left(a_{2}=x_{2},a_{1}=x_{1}\right)=$$

$$P_{C}\left(\omega \in \Omega :a_{1}\left(\omega \right)=x_{1},a_{2}\left(\omega \right)=x_{2}\right).$$

All observables show repeatability, since $P_{C}(a=x,a=x)=P_{C}(a=x),$ and RRE, since


$$P_{C}\left(a_{1}=x_{1},a_{2}=x_{2},a_{1}=x_{1}\right)=P_{C}\left(\omega \in \Omega :a_{1}\left(\omega \right)=x_{1},a_{2}\left(\omega \right)=x_{2},a_{1}\left(\omega \right)=x_{1}\right)=$$

$$P_{C}\,\left(a_{1}=x_{1},a_{2}=x_{2}\right).$$

The Bell inequalities are not violated, since their derivation is based on the existence of JPD.

We can summarize the properties of classical CMM by referring to the aforementioned list of possibilities:

– violation of FTP—no
– OE—no
– violation of replicability—no
– RRE—yes
– OE+RRE—yes
– violation of Bell inequalities—no

One of the problems of the above contextual representation of the classical probability is that the uniqueness conditions (2.2) and (2.3), Mackey’s axiom 2, can be violated, i.e. generally,


(3.5) $$P_{C}^{a_{1}}=P_{C}^{a_{2}}\;\text{ for any }C\in C⇏a_{1}=a_{2}.$$

(3.6) $$P_{C_{1}}^{a}=P_{C_{2}}^{a}\;\text{ for any }a\in O⇏C_{1}=C_{2}.$$

This problem can be easily resolved in the standard way (see below).

Let us consider a Kolmogorov probability space K=(Ω,F,P) with a complete probability measure, i.e. any subset of a set D∈F,P(D)=0, also belongs to F. We recall that the symmetric difference of two sets $D_{1}$ and $D_{2}$ is defined as


$$D_{1}\,\Delta \,D_{2}=\left(D_{1}\setminus D_{2}\right)\cup \left(D_{2}\setminus D_{1}\right)=\left(D_{1}\cup D_{2}\right)\setminus \left(D_{1}\cap D_{2}\right).$$

For $D_{1},D_{2}\in F,$ we set $D_{1}∼D_{2}$ if $P(D_{1}\Delta D_{2})=0.$ This is an equivalence relation on F; it splits F into disjoint equivalence classes. We denote the set of equivalence classes by the symbol $\tilde{F}$ and the equivalence class of zero probability sets by the symbol $\tilde{Z}.$ The set of pre-measurement contexts is $\tilde{C}=\tilde{F}\setminus \tilde{Z},$ i.e. all equivalent classes of sets from F of non-zero measure.

We also modify the class of observables. Two random variables are equivalent, $a_{1}∼a_{2},$ if $P(\omega \in \Omega :a_{1}(\omega )\neq a_{2}(\omega ))=0.$ This is the equivalence relation on the space of random variables; in our consideration, these are discrete random variables $R_{d}.$ So, $R_{d}$ is split into disjoint classes of equivalent random variables, and we denote the set of these classes by the symbol ${\tilde{R}}_{d}$ and set $\tilde{O}={\tilde{R}}_{d}.$

For a pre-measurement context $\tilde{C}\in \tilde{C}$ and observable $\tilde{a}\in {\tilde{R}}_{d}$ , we define the probability distribution $P_{\tilde{C}}^{\tilde{a}}(x)=P_{C}(a=x)$ for some representatives $C\in \tilde{C}$ and $a\in {\tilde{R}}_{d}$ (the correctness of this definition is proved below), and set $\tilde{P}=\{P_{\tilde{C}}^{\tilde{a}}\}.$ The modified classical contextual probability space is the triple $\tilde{\Sigma }=(\tilde{C},{\tilde{R}}_{d},\tilde{P}).$

The map $T_{a}(x),$ see equation (3.3), generates a map of $\tilde{F}$ into itself


(3.7) $${\tilde{T}}_{a}(x):\tilde{F}\to \tilde{F}.$$

Set ${\tilde{C}}_{a}(x)=\{\tilde{C}\in \tilde{C}:{\tilde{T}}_{a}(x)\tilde{C}\in \tilde{C}\}.$ Then, ${\tilde{T}}_{a}(x):{\tilde{C}}_{a}(x)\to \tilde{C}.$ A measurement context is a triple $(\tilde{C},\tilde{a},{\tilde{T}}_{a}).$ Modified classical CMM ${\tilde{M}}_{cl}$ is given by the set of such measurement contexts.

Now, we demonstrate that in ${\tilde{M}}_{cl}$ , the uniqueness conditions (2.2) and (2.3), Mackey’s axiom 2, hold true. We assume that the ranges of values of random variables are subsets of a set X; for simplicity, let X=R. First, we note that if $D_{1},D_{2}\in \tilde{D},$ then $P(D_{1})=P(D_{2}).$ We have $P(D_{1})=P((D_{1}\cap D_{2})\cup (D_{1}\setminus D_{2}))=P(D_{1}\cap D_{2})=P(D_{2}).$ Let now $C_{1},C_{2}\in \tilde{C},$ then, as we have seen, $P(C_{1})=P(C_{2});$ also, for any $G\in F,P(G\cap C_{1})=P(G\cap C_{1}\cap C_{2})=P(G\cap C_{2}).$ Hence, $P_{C_{1}}(G)=P_{C_{2}}(G).$ So, conditional probability measure $P_{C}$ does not depend on the choice of a representative $C\in \tilde{C}$ , and it can be denoted as $P_{\tilde{C}}.$

We show that implication (2.2) holds. Let for random variables $a_{1},a_{2},P_{C}^{a_{1}}=P_{C}^{a_{2}}$ for any context C. Let, for some $x,P(\Omega_{a_{1}=x})>0.$ Take $C=\Omega_{a_{1}=x},$ then


$$1=P_{\Omega_{a_{1}=x}}\,\left(\Omega_{a_{1}=x}\right)=P\,\left(\Omega_{a_{2}=x}\cap \Omega_{a_{1}=x}\right)/P\,\left(\Omega_{a_{1}=x}\right),$$

i.e.


$$P\left(\Omega_{a_{2}=x}\cap \Omega_{a_{1}=x}\right)=P\left(\Omega_{a_{1}=x}\right)=P(\left(\Omega_{a_{2}=x}\cap \Omega_{a_{1}=x}\right)\cup \left(\Omega_{a_{1}=x}\setminus \Omega_{a_{2}=x}\right).$$

Hence, $P(\Omega_{a_{1}=x}\setminus \Omega_{a_{2}=x})=0$ and symmetrically $P(\Omega_{a_{2}=x}\setminus \Omega_{a_{1}=x})=0.$ The sets $\Omega_{a_{i}=x},i=1,2,$ belong to the same equivalence class. This implies that the random variables also belong to the same equivalence class.

Now, we turn to implication (2.3). Let, for any random variable $a,P_{C_{1}}^{a}=P_{C_{2}}^{a}.$ select a as the characteristic function of the set $C_{1}.$ Then, $P_{C_{1}}^{a}(1)=1=P(C_{1}\cap C_{2})/P(C_{2}),$ i.e. $P(C_{1}\cap C_{2})=P(C_{2})=P((C_{1}\cap C_{2})\cup (C_{2}\setminus C_{1}),$ i.e. $P(C_{2}\setminus C_{1})=0$ and symmetrically $P(C_{1}\setminus C_{2})=0,$ i.e. contexts belong to the same equivalence class.

## 4. Contextual measurement model for von Neumann observables

We restrict the consideration to finite-dimensional Hilbert state spaces. The space of pre-measurement contexts is mathematically represented as the space of density operators D , i.e. C=D, and observables are Hermitian operators (von Neumann observables [1,37]). We denote the space of Hermitian operators by the symbol $L_{H},$ i.e. $O=L_{H}.$ This real linear space is endowed the with scalar product $\langle \hat{A}|\hat{B}\rangle =Tr\hat{A}\hat{B}.$

The operator $\hat{A}\in L_{H}$ has the spectral decomposition: $\hat{A}=\sum_{x\in X_{A}}x\;{\hat{E}}_{A}(x),$ where ${\hat{E}}_{A}(x)$ is the orthogonal projection on the subspace $H_{A}(x)$ composed of eigenvectors with eigenvalue x, and $X_{A}$ is operator’s spectral set. Then,


(4.1) $$P_{\rho }^{A}(x)\equiv P_{\rho }(A=x)=Tr{\hat{E}}_{A}(x)\hat{\rho }=Tr{\hat{E}}_{A}(x)\hat{\rho }{\hat{E}}_{A}(x),$$

(4.2) $$T_{A}(x):D\to D,{\hat{\rho }}_{A=x}\equiv T_{A}(x)\hat{\rho }=\frac{{\hat{E}}_{A}(x)\hat{\rho }{\hat{E}}_{A}(x)}{Tr{\hat{E}}_{A}(x)\hat{\rho }{\hat{E}}_{A}(x)},$$

with the domain of definition $C_{A}(x)=\{\hat{\rho }\in D:P_{\rho }(A=x)>0\}.$

Thus, $P=\{P_{\rho }^{A}:\rho \in D,A\in L_{H}\}$ and $T=\{T_{A}(x):A\in L_{H},x\in X_{A}\}.$ We remark that observable A uniquely determines the family of maps $T_{A}(x),x\in X_{A}$ by equality (4.2). So, measurement contexts can be represented by pairs $(\hat{\rho },\hat{A}),\hat{\rho }\in D,\hat{A}\in L_{H}.$ We denote this CMM by the symbol $M_{QVN}.$

In this CMM, one need not define separately the context update maps, they are automatically encoded in observables. On the one hand, this simplifies theory. On the other hand, this is the misleading path in measurement theory, cf. with quantum instrument theory.

In CMM $M_{QVN},$ the conditional probability is given by the formula


(4.3) $$P_{\rho }(B=y|A=x)=Tr{\hat{E}}_{B}(y)T_{A}(x)\hat{\rho }=\frac{Tr{\hat{E}}_{B}(y){\hat{E}}_{A}(x)\hat{\rho }{\hat{E}}_{A}(x)}{Tr{\hat{E}}_{A}(x)\hat{\rho }{\hat{E}}_{A}(x)}.$$

It can be rewritten as


(4.4) $$P_{\rho }(B=y|A=x)=\frac{Tr{\hat{E}}_{B}(y){\hat{E}}_{A}(x)\hat{\rho }{\hat{E}}_{A}(x){\hat{E}}_{B}(y)}{Tr{\hat{E}}_{A}(x)\hat{\rho }{\hat{E}}_{A}(x)}.$$

In this LSR-based CMM, it is convenient to introduce the maps:


(4.5) $$I_{A}(x)\hat{\rho }={\hat{E}}_{A}(x)\hat{\rho }{\hat{E}}_{A}(x).$$

Then, the above formulas can be rewritten as


(4.6) $$P_{\rho }(A=x)=TrI_{A}(x)\hat{\rho },$$

(4.7) $$T_{A}(x)\hat{\rho }=\frac{1}{P_{\rho }(A=x)}I_{A}(x)\hat{\rho }$$

or


(4.8) $$I_{A}(x)\hat{\rho }=P_{\rho }(A=x)T_{A}(x)\hat{\rho };$$

and the conditional probability is written in the form


(4.9) $$P_{\rho }(B=y|A=x)=\frac{TrI_{B}(y)I_{A}(x)\hat{\rho }}{TrI_{A}(x)\hat{\rho }},$$

and conditional JPD as


(4.10) $$P_{\rho }(A=x,B=y)=TrI_{B}(y)I_{A}(x)\hat{\rho }.$$

These formulas lead to the quantum instrument theory (§5): $\left(A,I_{A}(x)\right)$ is a special quantum instrument, and $\left(A,T_{A}(x)\right)$ is the corresponding contextual instrument.

We note that in CMM $M_{QVN}$ probabilities determine contexts (states) and observables (operators), i.e. equations (2.2) and (2.3) hold (Mackey’s axiom 2).

Let $P_{\rho }^{A_{1}}(x)=P_{\rho }^{A_{1}}(x)$ for all $\hat{\rho }\in D$ and real x. Then, $Tr(E_{A_{1}}(x)-E_{A_{2}}(x))\hat{\rho }=\langle E_{A_{1}}(x)-E_{A_{2}}(x)|\hat{\rho }\rangle =0.$ Hence, $E_{A_{1}}(x)=E_{A_{2}}(x)$ for any x; so ${\hat{A}}_{1}={\hat{A}}_{2}.$ Thus, an observable can be identified with the set of probability distributions $P_{A}=\{P_{\rho }^{A}:\hat{\rho }\in D\}.$

Now let $P_{\rho_{1}}^{A}(x)=P_{\rho_{2}}^{A}(x)$ for all $\hat{A}\in L_{H}$ and real x. Then, $TrE_{A}(x)({\hat{\rho }}_{1}-{\hat{\rho }}_{2})=\langle E_{A}(x)|{\hat{\rho }}_{1}-{\hat{\rho }}_{2}\rangle =0.$ Hence, ${\hat{\rho }}_{1}={\hat{\rho }}_{2}.$ Thus, a quantum state can be identified with the set of probability distributions $P_{\rho }=\{P_{\rho }^{A}:A\in L_{H}\}.$

In the von Neumann measurement theory, two observables are *compatible* if they are represented by commuting operators $\hat{A},\hat{B}:\;[\hat{A},\hat{B}]=0.$ Compatibility is interpreted as guaranteeing the possibility of joint measurement of these observables; their JPD is given by the formula:


(4.11) $$P_{\rho }^{A,B}=Tr{\hat{E}}_{A}(x){\hat{E}}_{B}(y)\rho =Tr{\hat{E}}_{B}(y){\hat{E}}_{A}(x)\rho .$$

In fact, this is the separate axiom—a complement to the Born rule [37]. For compatible observables, JPD and conditional JPD coincide. The conditional JPD is given by


(4.12) $$P_{\rho }(A=x,B=y)=P_{\rho }(A=x)P_{\rho }(B=y|A=x)=$$

$$P_{\rho }\left(A=x\right)P_{\rho_{A=x}}\left(B=y\right)==\mathrm{Tr}{\hat{E}}_{B}\left(y\right){\hat{E}}_{A}\left(x\right)\rho {\hat{E}}_{A}\left(x\right)=$$

$$\mathrm{Tr}\,{\hat{E}}_{A}\,\left(x\right)\,{\hat{E}}_{B}\,\left(y\right)\,{\hat{E}}_{A}\,\left(x\right)\,\rho =\mathrm{Tr}\,{\hat{E}}_{B}\,\left(y\right)\,{\hat{E}}_{A}\,\left(x\right)\,\rho .$$

In particular, for compatible observables, there is no OE for any state ρ. If operators ${\hat{A}}_{1},{\hat{A}}_{2}$ do not commute, then there exists a state $\hat{\rho }$ showing OE for these observables, i.e. $P_{\rho }(A=x,B=y)\neq P_{\rho }(B=y,A=x).$

Each observable A shows replicability, e.g.


(4.13) $$P_{\rho }(A=x,A=x)=Tr{\hat{E}}_{A}(x){\hat{E}}_{A}(x)\hat{\rho }{\hat{E}}_{A}(x)=Tr{\hat{E}}_{A}(x)\hat{\rho }=P_{\rho }(A=x).$$

If observables are compatible, then for any $\hat{\rho }$ , they show RRE, e.g.


$$P_{\rho }\,\left(A_{1}=x_{1},A_{2}=x_{2},A_{1}=x_{1}\right)=\mathrm{Tr}\,{\hat{E}}_{A_{1}}\,\left(x_{1}\right)\,{\hat{E}}_{A_{2}}\,\left(x_{2}\right)\,{\hat{E}}_{A_{1}}\,\left(x_{1}\right)\,\hat{\rho }\,{\hat{E}}_{A_{1}}\,\left(x\right)\,{\hat{E}}_{A_{2}}\,\left(x_{2}\right)=$$

$$\mathrm{Tr}\,{\hat{E}}_{A_{2}}\,\left(x_{2}\right)\,{\hat{E}}_{A_{1}}\,\left(x_{1}\right)\,\hat{\rho }\,{\hat{E}}_{A_{1}}\,\left(x\right)=P_{\rho }\,\left(A_{1}=x_{1},A_{2}=x_{2}\right).$$

We highlight that it is impossible to combine OE and RRE within CMM $M_{QVN}$ [60] (in the finite-dimensional case).

FTP can be violated; classical FTP is additively perturbed by the interference term; for instance, consider a pure state |ψ⟩, and then


(4.14) $$P_{\psi }(B=\beta )=\sum_{\alpha ,\alpha^{\prime }}\langle \psi |{\hat{E}}_{A}(\alpha ){\hat{E}}_{B}(\beta ){\hat{E}}_{A}(\alpha^{\prime })|\psi \rangle$$

$$=\sum_{\alpha }P_{\psi }(B=\beta |A=\alpha )P_{\psi }(A=\alpha )+\delta_{\psi }(B=y|A),$$

where


(4.15) $$\delta_{\psi }(B=y|A)=\sum_{\alpha \neq \alpha^{\prime }}\langle \psi |{\hat{E}}_{A}(\alpha ){\hat{E}}_{B}(\beta ){\hat{E}}_{A}(\alpha^{\prime })|\psi \rangle .$$

On the r.h.s., the first summand corresponds to classical FTP, and the second one is the interference term; it quantifies the degree of non-classicality for this CMM; see §2.3 for FTP in the general contextual probabilistic framework.

Consider dichotomous observables, $A=x_{1},x_{2}$ and $B=y_{1},y_{2},$ of the von Neumann type. In this case, the interference term has the form


$$\delta_{\psi }\,\left(B=y|A\right)=$$

(4.16) $$2\cos\theta \sqrt{P_{\psi }(B=y|A=x_{1})P_{\psi }(A=x_{1})P_{\psi }(B=y|A=x_{2})P_{\psi }(A=x_{2})},$$

where the angle θ=θ(B=y|A;ψ).

We can summarize the properties of von Neumann CMM with the list of possibilities presented above:

– iolation of FTP—yes (equations (4.14) and (4.15)).
– OE—yes. This is a straightforward consequence of the existence of incompatible observables represented by non-commuting operators.
– iolation of replicability—no (equation (4.13)).
– RRE—no. This is again a consequence of the incompatibility of some observables. Consider non-commuting operators $\hat{A}$ and $\hat{B}.$ For simplicity, assume that they have non-degenerate spectra; consider the orthonormal bases $\left(e_{j}^{A}\right)$ and $\left(e_{j}^{B}\right)$ consisting of operators’ eigenvectors with eigenvalues $\left(a_{j}\right)$ and $(b_{j}).$ At least for one pair of indexes $k,m,0<|\langle e_{k}^{A}|e_{m}^{B}\rangle |<1.$ Let the initial state be a pure state |ψ⟩. Suppose that the measurement of observable A gives the outcome $a_{k}$ and the sequential measurement observable B gives the outcome $b_{m}.$ Let observable A be measured again. Then, the probability to get again the outcome $a_{k},$ given by $|\langle e_{k}^{A}|e_{m}^{B}\rangle {|}^{2},$ does not equal one. The case of operators with degenerate spectra is studied in the article [60]. It is important to note that we consider only the finite-dimensional case.
– OE+RRE—no. Observables demonstrating OE should be incompatible. But, for incompatible observables, RRE is violated.
– Violation of Bell inequalities—yes [43,44].

## 5. Contextual measurement model for quantum instruments

In this section, we present CMM $M_{QI}$ , where a measurement process is mathematically described by quantum instrument theory [7,8,11,12,46–52]. This CMM extends CMM $M_{QVN}$ , where a measurement process is mathematically described by a Hermitian operator (von Neumann observable).

The space of linear Hermitian operators $L_{H}$ is a real Hilbert space. We consider linear operators acting in it to be *superoperators*. A superoperator T is called positive if it maps the set of positive semidefinite operators onto itself: for g≥0,T(g)≥0.

Consider an observable A with a finite range of values X. Its measurements can be performed with various apparatuses; for each apparatus, the corresponding measurement procedure is mathematically described in the following way.

Any map x→I(x), where for each x∈X, the map I(x) is a positive superoperator, and


(5.1) $$I(X)\equiv \sum_{x\in X}I(x):D\to D$$

is called a *quantum instrument*. It determines some observable; we denote it by the symbol A ; see equation (5.9).

The probability of the output A=x is given by the generalized Born rule in the form


(5.2) $$P_{\rho }(A=x)=Tr\;[I(x)\rho ].$$

We note that the measurement with the output A=x generates the state update through the transformation


(5.3) $$\rho \to \rho_{A=x}=T_{A}(x)\rho \equiv \frac{I(x)\rho }{TrI(x)\rho },$$

with the domain of definition $C_{A}(x)=\{\hat{\rho }\in D:P_{\rho }(A=x)>0\}.$

Let


(5.4) $$I(x)\rho =\hat{E}(x)\rho \hat{E}(x),$$

where $\left(\hat{E}(x)\right)$ are projections giving the orthogonal decomposition of I. Such an instrument is called a projection instrument.

The most natural generalization of projection instruments is an atomic instrument. Let $\left(\hat{V}(x)\right)$ be a family of linear operators constrained by the normalization condition:


(5.5) $$\sum_{x}\hat{V}(x){\hat{V}}^{⋆}(x)=I.$$

An atomic quantum instrument is a superoperator of the form


(5.6) $$\rho \to I(x)\rho =\hat{V}(x)\rho {\hat{V}}^{⋆}(x).$$

Applications of the quantum instrument theory to quantum information are typically restricted by the use of atomic instruments.

The space of Hermitian operators $L_{H}$ is the real Hilbert space, i.e. for each linear operator acting in $L_{H}$ (superoperator), its adjoint is well defined. The generalized Born rule can be written as


(5.7) $$P_{\rho }(A=x)=\langle I(x)\rho |I\rangle =\langle \rho |I^{⋆}(x)I\rangle =\langle I^{⋆}(x)I|\rho \rangle ,$$

where I is the unit operator and $I^{⋆}(x)$ is the superoperator that is adjoint to I(x) in Hilbert space $L_{H}.$ Hence, the generalized Born rule has the form


(5.8) $$P_{\rho }(A=x)=Tr\hat{A}(x)\rho ,$$

where


(5.9) $$\hat{A}(x)=I^{⋆}(x)I.$$

Operators $\hat{A}(x),x\in X$ are called *effects*; they are positive semi-definite Hermitian and sum up to the unit operator:


$$\sum_{x\in X}\hat{A}\,\left(x\right)=I.$$

The family of operators $A=(\hat{A}(x),x\in X)$ is called a POVM; for a subset Δ of X, we set


$$\hat{A}\,\left(\Delta \right)=\sum_{x\in \Delta }\hat{A}\,\left(x\right)\geq 0;$$

this is an additive operator-valued measure, i.e. for $\Delta_{1},\Delta_{2}\subset X,\Delta_{1}\cap \Delta_{2}=\emptyset ,$


$$\hat{A}\,\left(\Delta_{1}\cup \Delta_{2}\right)=\hat{A}\,\left(\Delta_{1}\right)+\hat{A}\,\left(\Delta_{2}\right).$$

Instruments of the projection type, equation (5.4), determine the special class of POVMs, *projection-valued measures* (PVMs).

Two POVMs $A=(\hat{A}(x),x\in X)$ and $B=(\hat{B}(y),y\in Y)$ are called *compatible*, if there exists a POVM $C=(\hat{C}(x,y),(x,y)\in X\times Y)$ such that


(5.10) $$\hat{A}(x)=\sum_{y\in Y}\hat{C}(x,y),\;\hat{B}(x)=\sum_{x\in X}\hat{C}(x,y).$$

The compatibility is interpreted as guarantying the possibility of the joint measurement of these observables, and their JPD is given by generalization of the Born rule for compatible von Neumann observables:


(5.11) $$P_{\rho }^{A,B}(x,y)=Tr\hat{C}(x,y)\hat{\rho }.$$

In the contextual probability space, contexts are mathematically represented by density operators (quantum states), C=D, and observables by POVMs (also known as generalized quantum observables), and the probability distributions are determined by the generalized Born rule, equation (5.2). CMM $M_{QI}$ is endowed by quantum instrument maps updating quantum states (contexts) due to the measurement feedback, equation (5.3).

Consider POVM $\hat{A}=(\hat{A}(x))$ and all quantum instruments generating it via (5.9). Then, the corresponding state (context) update maps are defined by equality (5.3). The same POVM, a generalized observable, can be coupled to a variety of such maps. Therefore, the commonly used approach highlighting POVMs as generalized observables is ambiguous. POVMs are just byproducts generated by quantum instruments.

Quantum instruments considered earlier were invented in the article [7] (see also monograph [8]), and these are *Davies–Levis instruments*, so $M_{QI}=M_{QI;DL}.$ In quantum information theory, one uses the special class of quantum instruments given by *completely positive maps* I(x); we denote the corresponding CMM $M_{QI;O},$ where I use the index ‘O’ to mention Masanao Ozawa who contributed so much to the theory of such quantum instruments [11,12,46–49]. It is commonly assumed that the instruments belonging to $M_{QI;DL}\setminus M_{QI;O}$ are non-physical. I debated this question with Masanao Ozawa, and he firmly stays on this position. As was proved by him, only completely positive instruments can be realized through the indirect measurement scheme [11]. This scheme is adequate for quantum measurement processes, and any deviation from this scheme is non-physical. Nevertheless, it might be that in quantum-like modelling, even instruments that are not completely positive can find applications. Such applications would lead to the modification of the indirect measurement scheme, may be through the consideration of non-unitary interactions.

We remark that instrument maps I(x) are linear in the Hilbert space $L_{H}.$ In terms of context (state) update, these maps can be written as


(5.12) $$I(x)=P_{\rho }(A=x)T_{A}(x).$$

Hence, in a quantum instrument, CMM scaling of the update map $T_{A}(x)$ by probability $P_{\rho }(A=x)$ is a linear map. Generally, LSR-CMM with the context (state) space C=D can be endowed with context (state) update maps such that scaling (5.12) can be nonlinear. CMM $M_{QI;NL}$ with nonlinear context update maps might be useful for quantum-like modelling. It is interesting to find concrete applications of $M_{QI;NL}.$ Of course, such applications would lead to the modification of the indirect measurement scheme.

We can summarize properties of quantum instrument CMM, both models $M_{QI;DL}$ and $M_{QI;O}.$ Since the quantum instrument model extends the von Neumann model, the majority of properties follow automatically from its properties.

– violation of FTP—yes
– OE—yes
– violation of replicability—yes
– RRE—no
– OE+RRE—generally no, but can be realized by special instruments
– violation of Bell inequalities—yes

So, $M_{QI}$ differs from $M_{QVN}$ with respect to replicability and OE+RRE combination. Replicability in $M_{QVN}$ is a consequence of projection state update. Such an update is idempotent, and thus the value of an observable is replicable. Model $M_{QI}$ permits more general updates; generally, they are not idempotent. The possibility to reproduce OE+RRE combination was demonstrated in the article [61]. This is a technically complicated construction within the theory of quantum instruments, and it is impossible to present this construction in the present paper.

It is interesting to find a property distinguishing $M_{QI;DL}$ and $M_{QI;O}$ through an experimental test, i.e. some experimentally testable property such that only completely positive instruments have it.

One of the important features of the von Neumann model is a coincidence of JPD and conditional JPD for compatible observables. In contrast, the instrument model shows that generally, the situation is not simple at all. Consider two instruments $I_{A}(x)$ and $I_{B}(y)$ such that their observables are POVMs of the PVM type, i.e. $\hat{A}=({\hat{E}}_{A}(x))$ and $\hat{B}=({\hat{E}}_{B}(y)).$ They are jointly measurable and the JPD is given by equation (4.11). The conditional JPD is given by


(5.13) $$P_{\rho }(A=x,B=y)=P_{\rho }(A=x)P_{\rho }(B=y|A=x)=$$

$$P_{\rho }\,\left(A=x\right)\,P_{\rho_{A=x}}\,\left(B=y\right)=\mathrm{Tr}\,{ℐ}_{B}\,\left(x\right)\,{ℐ}_{A}\,\left(x\right)\,\rho .$$

The r.h.s. of equations (4.11) and (5.13) coincide only if the instrument superoperators are of the projection type, i.e. $I(x)\rho =\hat{E}(x)\rho \hat{E}(x).$

Moreover, in this case, two projection-type observables, PVMs, can have a variety of conditional probability distributions corresponding to different instruments generating them by the rule $\hat{E}(x)=I^{⋆}(x)I.$

## 6. Ordered space measurement model with probability measure states

In this section, we connect the generalized probability theory (the Davies–Lewis approach [7]) for probability measures with CMM. Here, we use the ordered linear space approach. This is the concrete application of the universal scheme based on the abstract framework of ordered linear spaces.

Consider the space M of all real-valued measures on some set Ω with a σ -algebra of subsets F, i.e. M≡M(Ω,F). Real linear space M has the natural order structure and the positive cone $M^{+}$ consisting of non-negative measures. Consider the elements of this cone given by probability measures, i.e. μ≥0 and μ(Ω)=1; we denote this set by the symbol S; this is the set of states and S is a convex subset of M. The latter is endowed with the variation norm, ||μ||=var(μ) , and it is a Banach space. Consider its dual space $M^{\prime },$ the space of continuous linear functionals f:M→R. We denote by A the subset of $M^{\prime },$ consisting of functionals mapping S into [0,1]. Elements of A are called effects, and these are basic observables. They can be described solely in terms of the state space S as affine functionals valued in [0,1], i.e. A≡A(S).

Consider the functional $u\in M^{\prime }$ defined as μ→⟨u|μ⟩=μ(Ω). Its characteristic property is that ⟨u|μ⟩=1 for any state μ∈S.

Let $X=\{x_{1},..,x_{m}\}$ be a finite set and let $A=(A(x_{i}),i=1,...,m)$ where $A(x_{i})\in A(S)$ and $A(X)\equiv \sum_{x\in X}A(x)=u.$ Such vectors of functionals are analogues of POVMs; we call them M -POVMs. These are observables of the contextual probability space $\Sigma_{measure}$ with contexts C=S , and the set of probability distributions P defined as


$$P_{\mu }^{A}\,\left(x\right)\equiv P_{C}\,\left(A=x\right)=\left\langle A\,\left(x\right)|\mu \right\rangle .$$

As we learn from the quantum instrument theory, the basic elements of measurement procedures are not observables but instruments. Let L(M) denote the space of continuous linear operators, J:M→M. The M -instrument with the range of values X is a map I:X→L(M) such that $I(x)M^{+}\subset M^{+}$ and


$$ℐ\,\left(X\right)\equiv \sum_{x}ℐ\,\left(x\right):𝒮\to 𝒮.$$

Each instrument determines the state update map


$$\mu \to T_{A}(x)\mu \equiv \frac{1}{I(x)\mu (\Omega )}I(x)\mu =\frac{1}{\left\langle u|I(x)\mu \right\rangle }I(x)\mu ,$$

and the probability distribution


$$P_{\mu }(A=x)=\langle u|I(x)\mu \rangle .$$

The domain of definition of the state update map $T_{A}(x)$ is given by the set of probability measures $C_{A}(x)=\{\mu \in S:P_{\mu }(A=x)>0\}.$

Let J:M→M be a continuous linear operator. Then, its adjoint operator $J^{⋆}$ is well-defined, $J^{⋆}:M^{\prime }\to M^{\prime }$ and


$$\left\langle J^{⋆}\,f|\mu \right\rangle =\left\langle f|J\,\mu \right\rangle .$$

Set


$$A\,\left(x\right)={ℐ}^{⋆}\,\left(x\right)\,u.$$

Then, for each x,A(x) is an effect, i.e. A(x)∈A(S). So, each M -instrument determines a M -POVM.

The M -CMM consists of context (states) given by probability measures and POVM-observables with state updates given by instruments.

## 7. Linear space representation for contextual probability space

The state space is given by the set S , the set of possible measurement outcomes of an observable quantity is denoted by X. Let a system be in a state s∈S. A probability p(x,s) is assigned to any possible outcome x∈X. Thus, we have a function


p:X×S→[0,1].

To each outcome x∈X and state s∈S, this function is a probability of the outcome x for the system that is in the state s. The generalized probability model is a triple (S,p(⋅,⋅),X). We denote by $\Phi_{\left[0,1\right]}$ the space of function from X to [0,1]. By considering state s as a variable, we obtain the map


(7.1) $$S\to \Phi_{\left[0,1\right]},\;s\to s(\cdot )=p(\cdot ,s).$$

It is natural to assume that each state s∈S determines the probability distribution uniquely, i.e.


(7.2) $$p(x,s_{1})=p(x,s_{2})\;\text{ for any }x\in X\Rightarrow s_{1}=s_{2}.$$

Under this assumption, the map (7.1) is injection. Thus, each state s can be mapped to a function belonging to space $\Phi_{\left[0,1\right]},$ and it will be denoted by the same symbol s. Consider now the vector space Φ of all real-valued functions on X. So, S is identified with a subset of this functional space. Consider its closed convex hull $\bar{S}.$ The vectors from it are all possible probabilistic mixtures (convex combinations) of states in S.

Each x∈X defines a linear functional on $\Phi ,\;\phi \to f_{x}(\phi )=\phi (x).$ If $\phi =s\in \bar{S},$ then $f_{x}(s)=s(x)\in [0,1],$ i.e. $f_{x}:\bar{S}\to [0,1].$ This is an affine functional on the convex set $\bar{S}.$ It describes a measurement outcome, and $f_{x}(s)=p(x,s)$ is the probability for this outcome in state s.

We denote by $A(\bar{S})$ the space of all affine functionals


$$f:\bar{S}\to \left[0,1\right].$$

In particular, for any $x\in X,\;f_{x}\in A(\bar{S}).$ Any functional $f\in A(\bar{S})$ describes an outcome of some observable, and thus f(s) is the probability for that outcome in state s.

In QM, $\bar{S}$ is the set of density operators, and elements of $A(\bar{S})$ are called effects—components of POVMs, $f(\rho )=Tr{\hat{E}}_{f}\hat{\rho },$ where ${\hat{E}}_{f}$ is the effect corresponding to the affine functional f.

The elements of $A(\bar{S})$ are called *effects*. It is typically assumed that there exists an element u of $A(\bar{S})$ such that u(s)=1 for any $s\in \bar{S}.$ It is an analogue of quantum observable given by the unit operator I. Consider the point-wise order structure on $A(\bar{S}),f\leq g$ if f(s)≤g(s) for any state s. Thus, any observable $f\in A(\bar{S})$ is majorated by u,0≤f≤u. A discrete measurement is represented by a set of effects $\left(f_{i}\right)$ such that $\sum_{i}f_{i}=u.$

We now connect LSR to the contextual probabilistic model. We assume that all observables have the same range of values X. The straightforward intention is to set S=C×O . Let, as above, $\Phi_{\left[0,1\right]}$ denote the space of functions from X to [0,1]. We map S into $\Phi_{\left[0,1\right]},\;s=(C,A)\to P_{C}^{A}.$ However, generally, this map is not injection: $P_{C_{1}}^{A_{1}}(x)=P_{C_{2}}^{A_{2}}(x)$ for all x∈X does not imply that $C_{1}=C_{2}$ and $A_{1}=A_{2}.$ So, such straightforward construction seems to be non-proper for our aim.

We modify it by setting X=O×X, the elements of X are pairs x= (observable, outcome) =(A,x). We now use the symbols $\Phi_{\left[0,1\right]}$ and Φ for functions from X→[0,1] and to real line, respectively. Each context C can be represented as a vector belonging to $\Phi_{\left[0,1\right]},\;C(x)=C(A,x)=P_{C}^{A}(x).$ Due to equation (2.3), embedding of the set of contexts C into $\Phi_{\left[0,1\right]}$ is injection. Again, we denote by $\bar{C}$ the convex hull of C. Each point x= (observable, outcome) =(A,x) determines the affine functional $C\to f_{A,x}(C)=P_{C}^{A}(x)\in [0,1].$ Now fix A∈O and consider the family of functionals $F=(F_{A}(x)=f_{A,x}:x\in X).$ This is the representation of observable A.

So, any contextual probability model can be realized like COM—an observational COM.

## 8. Concluding remarks

As was emphasized in §1, CMM can be considered as the most general probabilistic model for measurement. It can also be considered as a minimalist restructuring of Mackey’s project [6]. Mackey proceeded to quantum logic, and this made the mathematical construction more complicated. One may even say that mathematics shadowed measurement theory. Surprisingly, even this minimalist model (CMM) has a complex structure and represents the basic elements of quantum probability and measurement theory, e.g. interference of probability, OE, entanglement and the violation of the Bell inequalities.

CMM can be employed not only in quantum foundations but also in quantum-like modelling that can employ contextual probability calculi and CMMs that are not based on the complex Hilbert space formalism.

Finally, we reproduce a list of the basic properties that can be used to classify CMMs:

– violation of FTP
– OE
– violation of replicability
– RRE
– OE+RRE
– violation of Bell inequalities.

Although two basic quantum measurement models, von Neumann’s model $M_{QVN}$ and the quantum instruments model $M_{QI},$ can be distinguished with respect to violation of replicability and OE+RRE combination, they both violate FTP, demonstrate OE and violate Bell inequalities. It would be interesting to find other CMMs that are distinguished, for example, with respect to the violation of FTP and Bell inequalities.

## Data Availability

This article has no additional data.

## Footnotes

^1^
Another approach to the generation of the complex Hilbert space representation of CMM is developed in a series of authors’ works (e.g. [29,54,55]). It is based on the contextual version of FTP, FTP with an interference term.

^2^
In applications outside of physics, in so-called quantum-like modelling, not all pre-measurement contexts can be straightforwardly represented in the form of a preparation procedure; here, we operate with mental, social and financial pre-measurement contexts. Within the statistical (ensemble) interpretation of QM, contexts are represented as ensembles of similarly prepared systems.

^3^
In terms of ensembles, *A*-measurement is performed for systems of initially prepared ensemble, and then systems generating the outcome *A*=*x* form new ensemble.

## Appendix A. Terminology: context versus state

We make the following remark about the terminology ‘context versus state’. Since QM operates with the notion ‘state’, generalized probability theory also employes this terminology. However, even in QM using the term ‘state’ is ambiguous. It matches the orthodox Copenhagen interpretation by which a state is treated as the state of an individual quantum system, say the state of an electron—one concrete electron. Many experts consider this interpretation of the quantum state as leading to paradoxes and mismatching with the statistical nature of quantum phenomena. This is a complicated foundational issue, since the leading supporters of the orthodox Copenhagen interpretation also consider QM as a statistical theory in which the state of an individual system encodes the statistics of the coming experimental runs.

For example, Einstein, Koopman, Margenau, Blohintzev and Ballentine and nowadays, for example, Ballian, Nieuwenhuizen and Khrennikov use the so-called statistical (or ensemble) interpretation of QM. By this interpretation, a quantum state represents statistical properties of an ensemble of identically prepared systems. So, whose state? The state of an ensemble? In the operational approach ‘state’ corresponds to a preparation procedure. It seems that the term ‘state’ borrowed from the orthodox Copenhagen interpretation does not match to the statistical and operational interpretations of QM. In the generalized probability theory, the term ‘state’ is typically associated with a preparation procedure or a class of equivalent preparation procedures. However, this meaning of the state is not highlighted, and the output of the generalized probability theory is often projected onto the orthodox Copenhagen interpretation, i.e. this theory interpreted as a theory about the structure of the state space of individual quantum systems. Therefore, in the Växjö interpretation, we prefer to use the notion of context as a complex of experimental conditions, and pre-measurement context can be associated with a class of equivalent preparation procedures (as is done in the consistent presentation of the generalized probability theory), and measurement context is the combination of the preparation, measurement and state update generated by measurement feedback with the fixed outcome.

In contrast to the generalized probability theory employing LSR, we do not assume that the set of pre-measurement contexts C contains contexts generated by statistical mixtures (see Axiom 4 in Mackey’s book [6]), i.e. for $C_{1},C_{2}\in C$ and $p_{1},p_{2}\geq 0,p_{1}+p_{2}=1,$ the set C need not contain a context that can be identified with $p_{1}C_{1}+p_{2}C_{2}.$ Proceeding without the mixture axiom illuminates the difference between the state and the context; consider, for example, the ‘basic contextual probability representation’ of the classical Kolmogorov probability space (§3). Here, contexts are not probability distributions but elements of the ( σ -)algebra. Generally, a context provides a finer description of the measurement setup than a probability distribution.

## Appendix B. Contextual measurement model with versus without linear space representation

### Why is it useful to proceed in contextual probabilistic framework as far as possible without appealing to linear space representation?

I start with some remarks on the uncritical use of LSR:

– LSR shadows the essence of the quantum probability formalism as the machinery for probability inference.
– LSR for classical probability, through the use of the linear space of measures with the positive cone of non-negative measures and convex state space of probability measures, seems to be inadequate to Kolmogorov’s theory [35,36] based on conditioning (contextualization) with Bayes’ formula (§3).
– LSR generates (through the creation of convex linear hull and its closure) a plenty of unphysical states and observables [18]), operating with them led, for instance, to von Neumann’s no-go theorem [37].^4^
– The picture that quantum probability theory is just one LSR of probability diminishes the exclusiveness of linearity in QM. One loses the physical ground for the latter, LSR becomes just a part of the mathematical apparatus of QM.
– Linking entanglement to the LSR tensor product structure shadows its contextual probabilistic nature and supports the ambiguous statements on quantum non-locality.
– Recently, the mathematical formalism of quantum theory, especially probability, started to be widely applied outside of physics, e.g. in cognition, psychology, social and political sciences, and economics and finance, the so-called quantum-like modelling (e.g. [39]). In such models, the set of possible states (pre-measurement contexts) is not as rich as in physics. In quantum-like modelling, even the possibility of preparing statistical mixtures is not evident, i.e. proceeding towards convex structures might be misleading.
